# Contrasting Magnetic Characteristics of Disordered Nd_0.5_Ba_0.5_Mn_0.5_Fe_0.5_O_3−δ/2_ and 112-Type Ordered NdBaMnFeO_6−δ_ Perovskites

**DOI:** 10.1021/acsomega.4c04607

**Published:** 2024-08-15

**Authors:** Aslam Hossain, Artem R. Gilev, Premakumar Yanda, Vladimir A. Cherepanov, Kathiresan Sakthipandi, A. Sundaresan, E. A. Mukhanova, Alexander V. Soldatov, A. K. M. Atique Ullah

**Affiliations:** † Smart Materials Research Institute, Southern Federal University, Sladkova 178/24, Rostov-on-Don 344090, Russia; ‡ Department of Physical and Inorganic Chemistry, Institute of Natural Sciences and Mathematics, Ural Federal University, Yekaterinburg 620026, Russia; § School of Advanced Materials and Chemistry and Physics of Materials Unit, Jawaharlal Nehru Centre for Advanced Scientific Research, Bangalore 560064, India; ∥ Department of Physics, SRM TRP Engineering College, Tiruchirappalli, Tamil Nadu 621 105, India; ⊥ Department of Chemistry, Michigan State University, East Lansing, Michigan 48824, United States

## Abstract

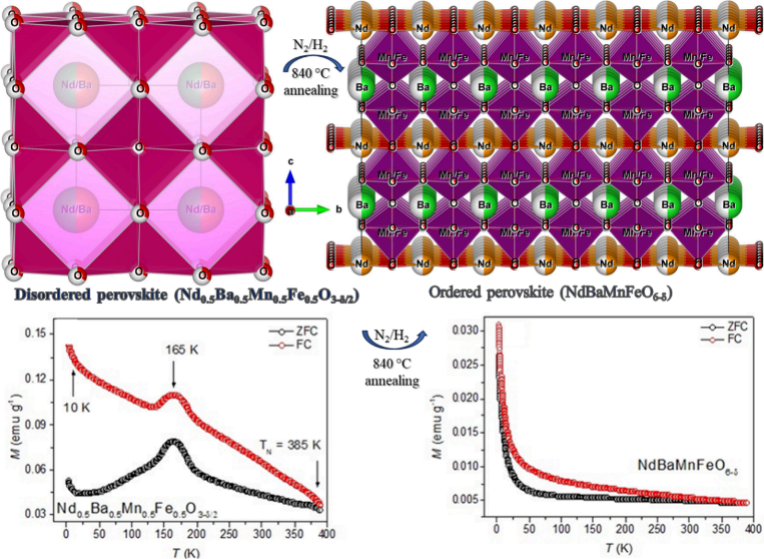

The magnetic properties of disordered Nd_0.5_Ba_0.5_Mn_0.5_Fe_0.5_O_3−δ/2_ and ordered NdBaMnFeO_6−δ_ perovskites were investigated through temperature- and field-dependent DC-magnetization measurements. The temperature dependence of magnetic susceptibilities revealed that antiferromagnetic ordering occurs at temperatures below 185 K for the disordered Nd_0.5_Ba_0.5_Mn_0.5_Fe_0.5_O_3-δ/2_ sample, whereas the ordered NdBaMnFeO_6−δ_ perovskite exhibited a paramagnetic state throughout the entire temperature range examined. Notably, the disordered sample exhibited a glassy state, even at room temperature, which transformed into an antiferromagnetic state under higher applied magnetic fields. The magnetic ordering in the disordered Nd_0.5_Ba_0.5_Mn_0.5_Fe_0.5_O_3-δ/2_ perovskite and the magnetic-disordering state in the structurally ordered NdBaMnFeO_6-δ_ perovskite could be attributed to the alteration of the oxidation state of Mn.

## 1 Introduction

Perovskite oxides are versatile materials known for their unique magnetic and electronic properties.^1^ They are widely studied for several modern applications due to their interesting properties.^2−4^ The study of Ln_0.5_Ba_0.5_MnO_3_ perovskites, where Ln represents a rare earth element, has garnered considerable interest from both the scientific and engineering communities. This interest is primarily due to the intriguing physical properties exhibited by these materials, which are a result of their A-site ordered-disordered structure.^5−8^ However, simple perovskite and corresponding A-site ordered double perovskites have attracted significant attention due to their promising applications in photovoltaics, photocatalysis, and microsensors for gas detection.^9−13^ Similarly, these materials also have been studied for their potential as cathode materials in solid oxide fuel cells (SOFCs) due to their mixed ionic-electronic conductivity and high catalytic activity for oxygen reduction reactions.^14,15^ The partial substitution of 3*d* metals can enhance the materials’ conductivity and stability at high temperatures, making it a promising candidate for efficient energy conversion. These double perovskite oxides, with a tetragonal structure and space group *P*4/*mmm*, exhibit diverse magnetic and electronic properties due to the interplay between structure, charge, and spin ordering.^16^ These properties make them valuable for spintronics, an emerging field with potential major impacts on electronics.^17^

The 112-type ordering of these perovskites induces a distortion in the MnO_6_ octahedra, which in turn influences the physical properties of the materials, including colossal magnetoresistance (CMR), charge ordering, orbital ordering, phase separation, and the Jahn–Teller effect.^18,19^ The Goodenough–Kanamori (GK) rule suggests a strong ferromagnetic Mn^3+^(d^4^)—O–Fe^3+^(d^5^) interaction in LaMn_0.5_Fe_0.5_O_3_. However, experimental observations have indicated a cluster-glass-like behavior with significant thermomagnetic irreversibility below 260 K.^20,21^ The compound LaMn_1–x_Fe_*x*_O_3_ (*x* ≤ 0.4) has been reported to exhibit ferromagnetism due to double exchange between Mn^3+^(d^4^) and Fe^3+^(d^5^). The transition temperature (from ferromagnetic to paramagnetic) gradually increases with barium doping of La_1–*x*_Ba_*x*_MnO_3_, reaching 339 K for La_0.5_Ba_0.5_MnO_3_.^22^ However, substituting lanthanum with neodymium reduces the magnetic transition temperature to approximately 80 K.^23^ NdMn_0.5_Fe_0.5_O_3_ has been reported to exhibit magnetic ordering with a G-type antiferromagnetic structure (slightly above room temperature) and spin-glass behavior at low temperatures.^23^

A-site ordered manganites prepared under reduction conditions have been found to require smaller magnetic fields to induce larger changes in electrical resistivity compared to disordered manganites synthesized in air.^24^ This has led to a 10-fold increase in negative magnetoresistance for ordered LnBaMn_2_O_6_ (Ln = Pr, Nd, and Sm) compared to the disordered variant.^24^ This ordering also results in an increase in the magnetic transition temperature, as evidenced by the increase in magnetic ordering temperature from 80 to 310 K in NdBaMn_2_O_6−δ_ due to the ordering of Nd^3+^ and Ba^2+^.^25^ The 112-type ordered NdBaMnFeO_6-δ_ perovskite was first synthesized via a topotactic reaction from the Nd_0.5_Ba_0.5_Mn_0.5_Fe_0.5_O_3−δ/2_ perovskite.^26^ The initial perovskite, Nd_0.5_Ba_0.5_Mn_0.5_Fe_0.5_O_3−δ/2_, exhibited a cubic structure with a space group (sp.gr.)  and a unit cell parameter *a* = 3.898(7) Å at room temperature. The resulting A-site ordered NdBaMnFeO_5.09_ perovskite possessed a tetragonal structure with sp. gr. *P*4/*mmm* and unit cell parameters *a* = 3.9889(1) Å and *c* = 7.6924(1) Å at room temperature. It was found that the maximum oxygen content in NdBaMnFeO_6−δ_ was limited to approximately 5.45, compared to 6 and 5.792 for NdBaMn_2_O_6−δ_ and NdBaFe_2_O_6−δ_, respectively, due to the higher distortion of the square-pyramidal/octahedral surrounding of 3d-metals.^26^

In this study, our objective is to investigate the magnetic properties of disordered Nd_0.5_Ba_0.5_Mn_0.5_Fe_0.5_O_3−δ/2_ and ordered NdBaMnFeO_6−δ_ perovskites with a specific focus on assessing the influence of A-site ordering on the magnetic behavior exhibited by these perovskite materials. The combustion technique is followed for synthesizing the materials due to its rapid and efficient process that reduces reaction times and produces highly pure and homogeneous powders. This method is also cost-effective, energy-efficient, and offers better control over particle size and morphology, making it suitable for both laboratory and industrial-scale production.^27−29^ By comparing the magnetic properties of the disordered and ordered perovskites, we aim to gain insights into the role of structural disorder and A-site ordering in modulating the magnetic characteristics of these materials. Such an investigation holds significance for understanding the interplay among structural distortions, magnetic ordering, and the resulting physical properties in complex perovskite systems.

## 2 Experimental Procedure

### 2.1 Synthesis

**Figure 1:**
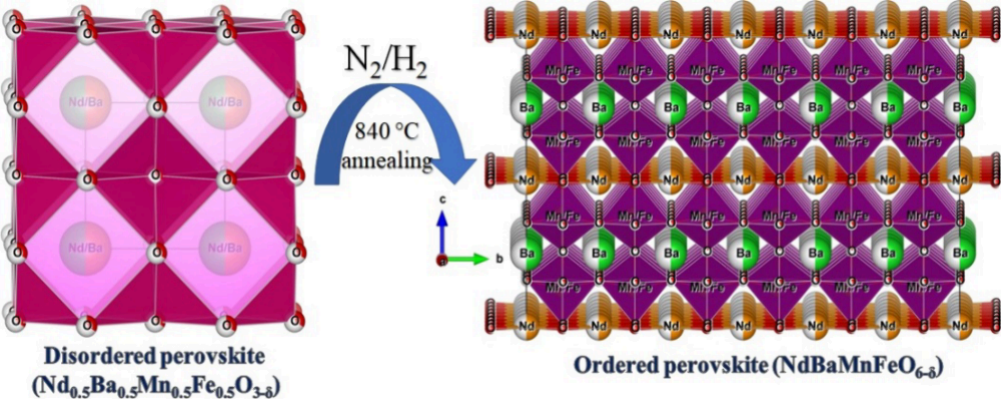
(Left) Crystal structure of disordered Nd_0.5_Ba_0.5_Mn_0.5_Fe_0.5_O_3-δ/2_; (right) ordered NdBaMnFeO_6−δ_ perovskite.

The synthesis of the disordered Nd_0.5_Ba_0.5_Mn_0.5_Fe_0.5_O_3−δ/2_ perovskite was achieved using the citrate-nitrate combustion technique, as described in previous studies.^30−32^ This method involves combustion of a citrate-nitrate gel to produce the desired compound. Subsequently, an A-site ordered NdBaMnFeO_6-δ_ sample was prepared by annealing the disordered Nd_0.5_Ba_0.5_Mn_0.5_Fe_0.5_O_3−δ/2_ powder (Figure 1). The annealing process was conducted at a temperature of 840 °C for a duration of 8 h. This was carried out in a controlled atmosphere of a N_2_/H_2_ gas mixture using a Netzsch STA 409 PC Luxx instrument. The samples were confirmed to be phase pure and cation stoichiometric, with the oxygen content determined as per the methods outlined in ref (26).

### 2.2 Characterizations

The phase compositions of both the disordered Nd_0.5_Ba_0.5_Mn_0.5_Fe_0.5_O_3−δ/2_ and the ordered NdBaMnFeO_6-δ_ samples were analyzed at room temperature using X-ray powder diffraction (XRD) on an XRD-7000 Maxima instrument (Shimadzu). The XRD analysis was performed using Cu–Kα radiation (fwhm ∼0.01° at 2θ). Field and temperature-dependent DC-magnetization measurements were performed using a Superconducting Quantum Interference Device (SQUID) from Quantum Design, USA.

## 3 Results and Discussion

**Figure 2:**
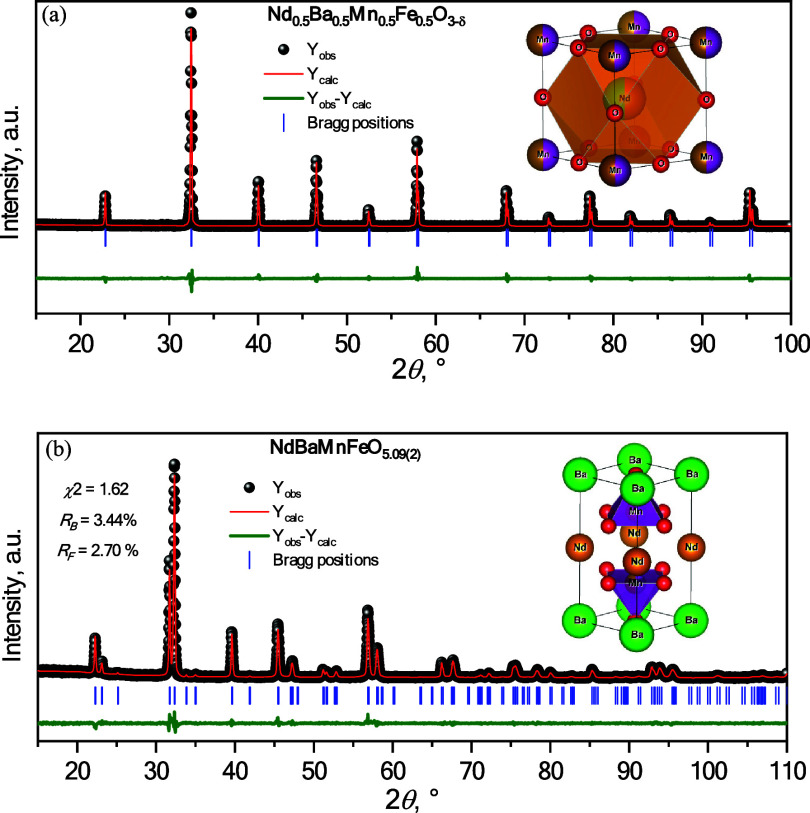
XRD patterns for (a) Nd_0.5_Ba_0.5_Mn_0.5_Fe_0.5_O_3−δ_ and (b) NdBaMnFeO_5.09_ refined by the Rietveld method. Inset shows their corresponding crystal structure.

The XRD patterns for Nd_0.5_Ba_0.5_Mn_0.5_Fe_0.5_O_3−δ_ and NdBaMnFeO_5.09_ perovskites refined by the Rietveld method are shown in Figure 2. Detailed characterization of the materials is reported elsewhere.^26^

**Figure 3:**
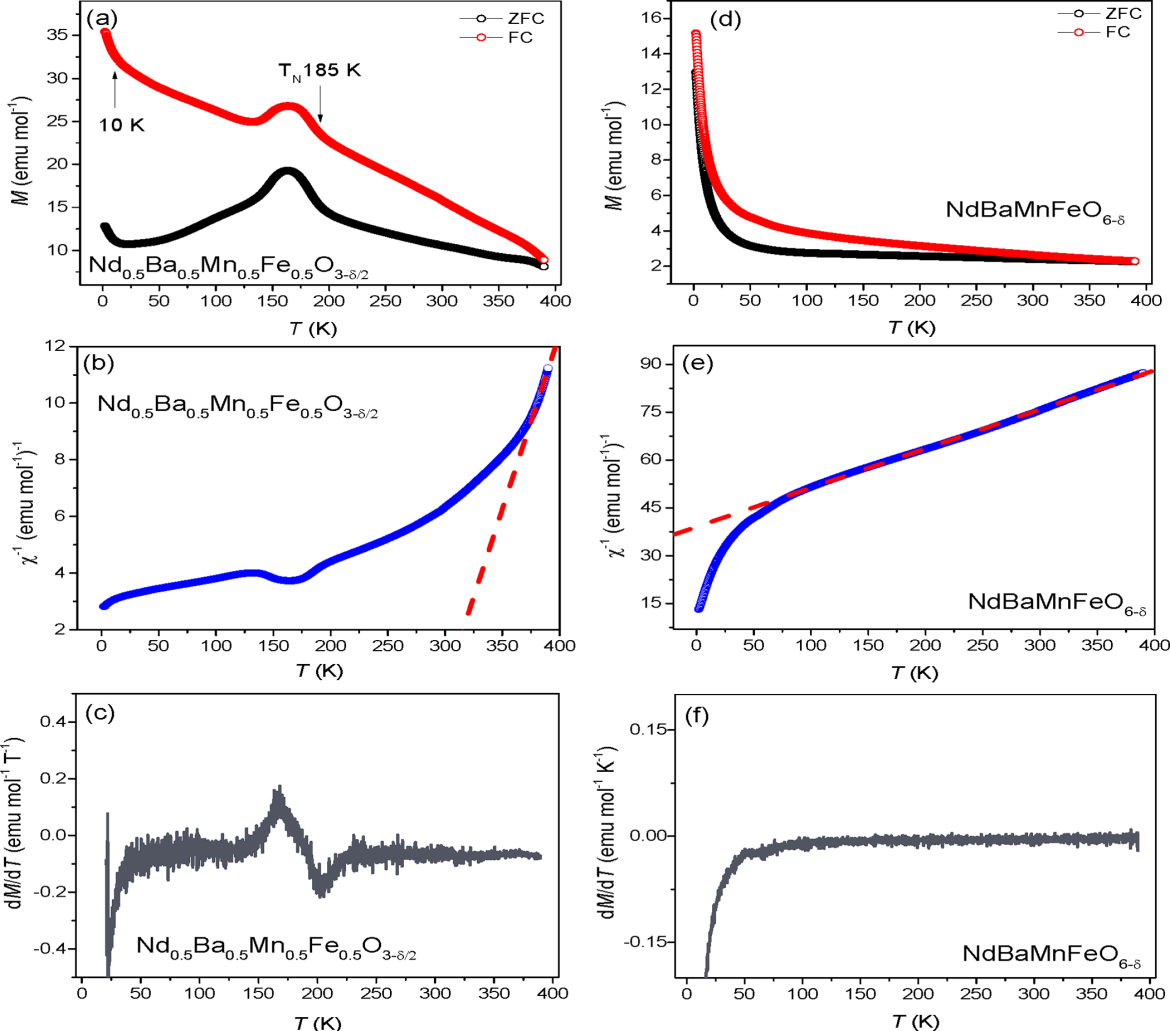
Temperature-dependent magnetization of disordered Nd_0.5_Ba_0.5_Mn_0.5_Fe_0.5_O_3-δ/2_ perovskite (a) *M*(*T*), (b) χ^–1^ (*T*), and (c) d*M*/d*T* (*T*) and ordered NdBaMnFeO_6-δ_ perovskite (d) *M*(*T*), (e) χ^–1^ (*T*), and (f) d*M*/d*T* (*T*).

The DC magnetization data, as a function of temperature, for Nd_0.5_Ba_0.5_Mn_0.5_Fe_0.5_O_3−δ/2_ and NdBaMnFeO_6−δ_, measured under an applied magnetic field of 100 Oe and within the temperature range of 2–390 K, is presented in Figure 3. The data was collected under both zero-field cooled (ZFC) and field cooled (FC) conditions. For Nd_0.5_Ba_0.5_Mn_0.5_Fe_0.5_O_3-δ/2_, two distinct anomalies were observed at 10 and 185 K and are indicative of a complex magnetic nature. The anomaly at 185 K is associated with antiferromagnetic ordering, as depicted in Figure 3b. At high temperatures, superexchange interactions between various cations are possible such as Fe–Fe, Mn–Mn, and Fe–Mn. The competition among these various interactions can lead to antiferromagnetic or spin glass magnetism. Here, an interesting point to refer to is that NdFeO_3_, BaMnO_3_, NdMn_0.5_Fe_0.5_O_3_, and Nd_0.5_Ba_0.5_MnO_3_ compounds exhibit antiferromagnetic ordering in the temperature range *T*_N_ = 200–260 K.^20,21^ Therefore, it can be plausible that the title compound also can exhibit antiferromagnetic ordering around 185 K. The reduction in the transition temperature can be expected as a consequence of competing interactions between Fe^3+^, Mn^3+^, and Mn^4+^. Further, spin reorientation transition is possible at a low temperature of 10 K upon the magnetic ordering of Nd^3+^ spins. Previously, similar behavior was reported in rhombohedral Bi_0.5_Sr_0.5_Fe_0.5_Mn_0.5_O_3_ where glassy state appeared at *T* < *T*_N_ = 226 K.^33^ A large thermomagnetic irreversibility in ZFC-FC data for Nd_0.5_Ba_0.5_Mn_0.5_Fe_0.5_O_3−δ/2_ is evident even at *T* > 300 K, as shown in Figure 3a suggests the presence of an inhomogeneous magnetic state. The observed behavior may be attributed to the coexistence of competing antiferromagnetic and weak ferromagnetic interactions due to the random substitution of Mn^4+^, Mn^3+^, and Fe^3+^ ions at the B-site.^23^

The susceptibility of the material above the Curie temperature (*T*_C_) follows the Curie–Weiss law, as shown in Figure 3b. The positive sign of the Weiss constant indicates weak ferromagnetic interactions between Fe^3+^ and Mn^4+^ ions. The behavior of the ZFC and FC data above 350 K suggests the presence of transition/additional magnetic phenomena occurring in the material at higher temperatures. The Weiss constant and the experimental effective paramagnetic moment for Nd_0.5_Ba_0.5_Mn_0.5_Fe_0.5_O_3−δ/2_ were determined to be approximately θ_*CW*_ ≈ 300 K and μ_*eff*_^*exp*^ = 8.04 μ_B_, respectively. To estimate the theoretical magnetic moment, the oxygen content value reported in ref (31) for the same sample was used, assuming that the majority of iron cations are in the Fe^3+^ state. Based on this, the chemical formula of the oxide at room temperature can be written as *Nd*_0.5_^3+^*Ba*_0.5_^2+^*Fe*_0.5_^3+^*Mn*_0.28_^3+^*Mn*_0.22_^4+^*O*_2.86_^2–^. The calculated value of the magnetic moment (μ_*eff*_^*th*^) was found to be 7.12 μB. The experimental magnetic moment is higher than the theoretical value, which could be attributed to short-range interactions between ferromagnetic clusters above *T*_C_.^34^ Another possible reason for the discrepancy is the fitting procedure conducted near the transition temperature. Additionally, the anomaly observed at 10 K is associated with the antiferromagnetic ordering of rare earth Nd^3+^ ions.

The ZFC and FC data for the ordered NdBaMnFeO_6−δ_ perovskite are shown in Figure 3d. The observed bifurcation between the ZFC and FC curves can be attributed to the presence of rare-earth Nd^3+^ ions. The behavior depicted in Figure 3d suggests the absence of interactions between magnetic clusters and the existence of a paramagnetic state at temperatures above 50 K. Figure 3e demonstrates that the magnetic susceptibility of NdBaMnFeO_5.09_ follows the Curie–Weiss law above 50 K. The negative Weiss constant (θ ≈ −318 K) indicates the possibility of dominant antiferromagnetic interactions. The effective paramagnetic moment for NdBaMnFeO_5.09_ is measured to be 8.07 μB, which closely aligns with the theoretical value (μ_*eff*_^*th*^ = 7.76 μ_B_). Significant changes in the dM/dT derivatives below 50 K, as observed in both the ordered and disordered samples (Figure 3c,f), suggest the antiferromagnetic ordering of rare-earth Nd^3+^ ions.^35^

**Figure 4:**
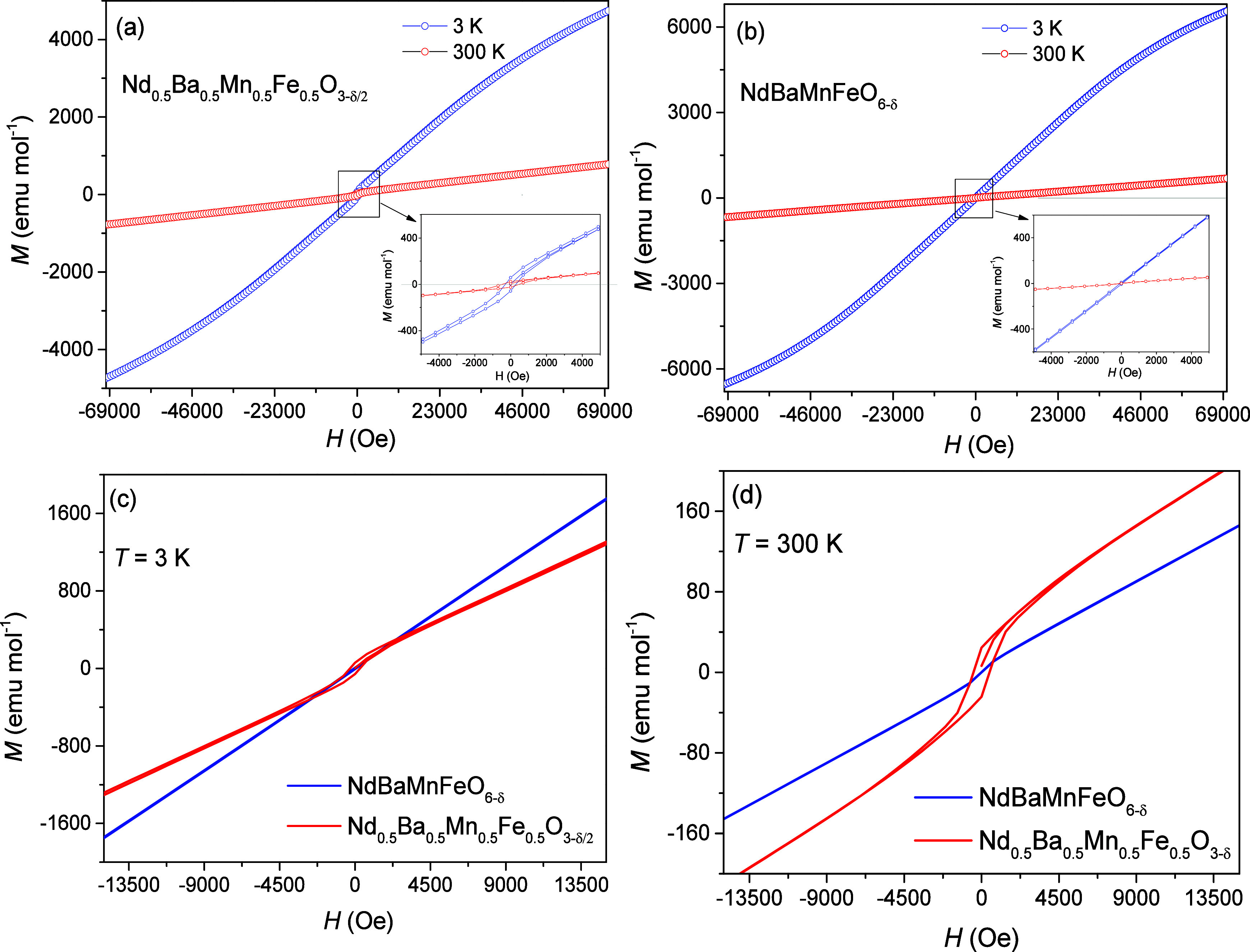
M–H curves for (a) Nd_0.5_Ba_0.5_Mn_0.5_Fe_0.5_O_3−δ/2_ and (b) NdBaMnFeO_6−δ_ at (c) 3 and (d) 300 K.

Figure 4 illustrates the field dependence magnetization of the synthesized samples, measured at both 3 and 300 K. The M vs H curves in Figure 4a display a linear behavior, indicative of antiferromagnetic ordering in the disordered Nd_0.5_Ba_0.5_Mn_0.5_Fe_0.5_O_3−δ/2_ sample, in line with previous studies.^34^ The shape of the M – H curves for this sample suggests a glassy state at lower fields. However, this glassy feature disappears at higher fields, presumably due to the forced alignment of all the frozen moments toward the externally applied magnetic field, demonstrating the influence of external fields on the magnetic state of the sample. At 3 K, the observed nonlinear behavior can be attributed to the meta magnetism of Nd ions. At 300 K, the hysteresis behavior of the disordered sample supports the ferromagnetic-paramagnetic transition, as detected from temperature-dependent magnetization (Figure 4d).

**Figure 5:**
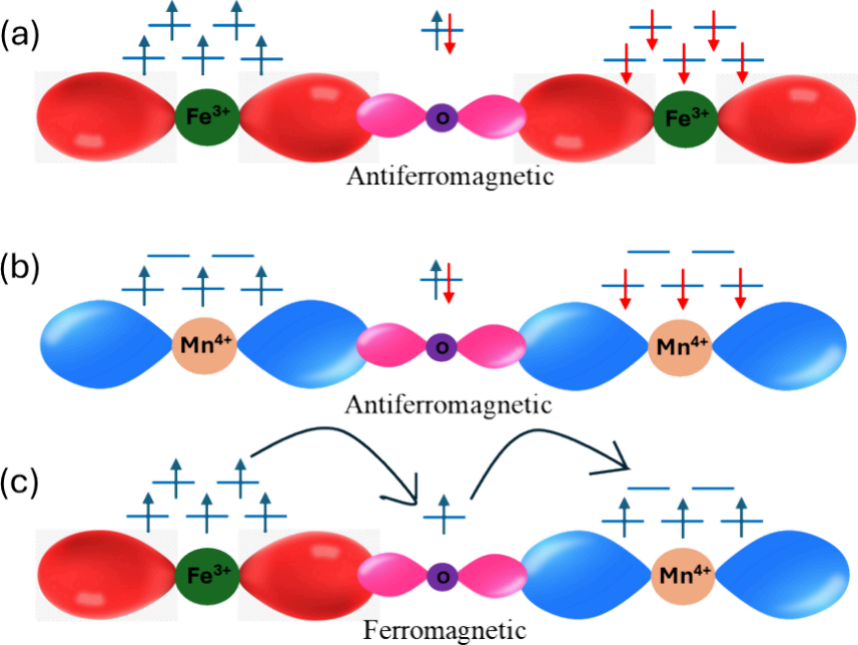
Schematic illustration showing (a, b) superexchange interactions favoring antiferromagnetic coupling and (c) double exchange interaction favoring ferromagnetic coupling between Fe and Mn ions, which is mediated via O ion lying between Fe and Mn.

According to the iodometric titration results reported in ref (26), the calculated average oxidation state of 3*d*-metals in Nd_0.5_Ba_0.5_Mn_0.5_Fe_0.5_O_3−δ/2_ is 3.22. If we assume the oxidation state of iron as Fe^3+^, then the ratio of Mn^3+^ and Mn^4+^ is approximately 4:3. In this case, the possible interactions for Nd_0.5_Ba_0.5_Mn_0.5_Fe_0.5_O_3−δ/2_ are Fe^3+^–O–Mn^3+^, Mn^3+^–O–Mn^4+^, Fe^3+^–O–Fe^3+^, Mn^3+^–O–Mn^3+^, and Fe^3+^–O–Mn^4+^. According to the GK rule, Fe–O–Fe and Mn–O–Mn super exchange interactions are antiferromagnetic, whereas Fe–O–Mn is ferromagnetic. The competition between these magnetic interactions can be illustrated by the Figure 5. This dichotomy of magnetic interactions suggests a potential competition, which could be responsible for the observed glassy nature in the Nd_0.5_Ba_0.5_Mn_0.5_Fe_0.5_O_3-δ/2_ compound.^36^ Further studies could focus on understanding the dynamics of these competing interactions and their role in the overall magnetic behavior of the compound.

The M–H curves for the ordered NdBaMnFeO_6−δ_ perovskite, as shown in Figure 4b, reveal the absence of a hysteresis loop even at 3 K, suggesting a paramagnetic state of spin. This finding aligns well with the data reported for La_0.5_Ba_0.5_Fe_0.5_Mn_0.5_O_3_.^6^ This suggests that the structural order in the perovskite influences its magnetic properties, leading to a paramagnetic state. Iodometric titration results indicate that the average oxidation state for NdBaMnFeO_6-δ_ is 2.59.^31^ Assuming that iron is in the Fe^3+^ state, the majority of the manganese cations appear to be in the +2 oxidation state for NdBaMnFeO_6−δ_, as inferred from the titration results. This suggests that the reduction of Mn^4+^ and Mn^3+^ to Mn^2+^ could be responsible for the differing magnetic behaviors observed between disordered Nd_0.5_Ba_0.5_Mn_0.5_Fe_0.5_O_3δ/2_ and structurally ordered NdBaMnFeO_6−δ_ perovskite.

## 4 Conclusions

The temperature-dependent magnetic susceptibilities revealed that the disordered Nd_0.5_Ba_0.5_Mn_0.5_Fe_0.5_O_3-δ/2_ perovskite exhibited antiferromagnetic ordering below temperatures of *T* < 185 K, whereas the ordered NdBaMnFeO_6-δ_ perovskite remained in a paramagnetic state across the entire temperature range investigated. The ZFC-FC data for Nd_0.5_Ba_0.5_Mn_0.5_Fe_0.5_O_3−δ/2_ indicated thermoirreversibility at temperatures below 385 K, suggesting a glassy nature arising from frustrated interactions between the ferromagnetic and antiferromagnetic states. The linear behavior observed in the M-H curves further supported the presence of antiferromagnetic ordering. However, additional comprehensive investigations are necessary to validate the occurrence of antiferromagnetic ordering and to characterize the complex spin glass behavior exhibited by the disordered compound. Future studies should aim to provide a more detailed understanding of the magnetic properties and underlying mechanisms in these materials, potentially through advanced characterization techniques and theoretical modeling.
